# Wastewater Levels of Respiratory Syncytial Virus Associated with Influenza-like Illness Rates in Children—A Case Study in Larissa, Greece (October 2022–January 2023)

**DOI:** 10.3390/ijerph20065219

**Published:** 2023-03-22

**Authors:** Michalis Koureas, Kassiani Mellou, Alexandros Vontas, Maria Kyritsi, Ioannis Panagoulias, Anastasia Koutsolioutsou, Varvara A. Mouchtouri, Matthaios Speletas, Dimitrios Paraskevis, Christos Hadjichristodoulou

**Affiliations:** 1 Laboratory of Hygiene and Epidemiology, Faculty of Medicine, University of Thessaly, 22 Papakyriazi Str., 41222 Larissa, Greece; 2 National Public Health Organization, 15123 Athens, Greece; 3 Department of Immunology and Histocompatibility, Faculty of Medicine, University of Thessaly, 41500 Larissa, Greece

**Keywords:** respiratory syncytial virus, wastewater-based epidemiology (WBE), SARS-CoV-2, influenza-like illness, RT-PCR

## Abstract

The emergence of the COVID-19 pandemic has led to significant progress in the field of wastewater-based surveillance (WBS) of respiratory pathogens and highlighted its potential for a wider application in public health surveillance. This study aimed to evaluate whether monitoring of respiratory syncytial virus (RSV) in wastewater can provide a comprehensive picture of disease transmission at the community level. The study was conducted in Larissa (Central Greece) between October 2022 and January 2023. Forty-six wastewater samples were collected from the inlet of the wastewater treatment plant of Larissa and analyzed with a real-time reverse transcription polymerase chain reaction (RT-PCR) based method. RSV and SARS-CoV-2 wastewater viral loads (genome copies/100,000 inhabitants) were analyzed against sentinel surveillance data on influenza-like illness (ILI) to identify potential associations. Univariate linear regression analysis revealed that RSV wastewater viral load (lagged by one week) and ILI notification rates in children up to 14 years old were strongly associated (std. Beta: 0.73 (95% CI: 0.31–1.14), *p* = 0.002, R^2^ = 0.308). A weaker association was found between SARS-CoV-2 viral load and ILI rates in the 15+ age group (std. Beta: 0.56 (95% CI: 0.06–1.05), *p* = 0.032, R^2^ = 0.527). The results support the incorporation of RSV monitoring into existing wastewater-based surveillance systems.

## 1. Introduction

Respiratory syncytial virus (RSV) is a single-stranded RNA virus of the Paramyxoviridae family, which is considered a leading cause of lower respiratory tract illness among young children [1]. A recent review estimated that in 2019, 33 million RSV-associated acute lower respiratory infections occurred globally, leading to 3.6 million hospital admissions and 101,400 RSV-attributable deaths in children younger than 5 years old [2]. RSV is inherently seasonal, and in temperate climates, it typically circulates during the fall and winter months [3]. The virus is primarily spread through respiratory droplets and has an incubation period between 2 and 8 days [4]. Most EU/EEA countries implement RSV surveillance, but there is a need to address issues concerning under-reporting and heterogeneity in RSV surveillance methods [5].

Wastewater–based surveillance (WBS) can strengthen existing RSV surveillance systems as it has certain advantages in terms of cost-effectiveness, speed and no sampling or reporting bias compared to surveillance based on individual testing. WBS is a method for acquiring information from wastewater samples to monitor the health status of a community. The concept of WBS in the field of infectious diseases is based on the fact that individuals excrete pathogen particles, which are carried through the sewerage system to central wastewater treatment plants (WWTPs). The analysis of samples collected from WWTPs can reflect the viral load of the entire population served by the sewerage system. The emergence of the COVID-19 pandemic has led to rapid and substantial advances in the field of WBS of respiratory pathogens, and many countries and institutions perform routine sewage monitoring of SARS-CoV-2 [6]. This implementation of WBS has naturally led to the need to explore its potential for public health surveillance beyond SARS-CoV-2 monitoring. The European Commission’s proposal for the new Urban Wastewater Treatment Directive clearly suggested the inclusion of more pathogens in urban wastewater surveillance systems [7]. The present study aims to examine whether monitoring of RSV in wastewater can potentially provide a comprehensive picture of disease transmission in a community by evaluating systematic wastewater sampling and analysis in combination with sentinel surveillance data on influenza-like illness (ILI).

## 2. Materials and Methods

Wastewater samples were collected from the WWTP of the municipality of Larissa, Central Greece. The sewerage system is 516 km long and only processes municipal wastewater. It serves approximately 150,000 residents. According to 2011 census data, approximately 17% of the population of the municipality of Larissa are children up to 14 years old. Twenty-four-hour composite samples were collected from the inlet of the plant with a Sigma SD900 portable sampler (HACH Company, Colorado, USA) at a rate of 150 mL per hour. After sampling, wastewater samples were transported to the laboratory in isothermal boxes at 2–8 °C. Sample pre-processing and RNA extraction were performed in accordance with the protocol for SARS-CoV-2 analysis that has been previously described [8]. The SuperScript™ III Platinum™ One-Step qRT-PCR Kit (Applied Biosystems™, Thermo Fisher Scientific, Waltham, MA, USA) was used for the real-time reverse transcription polymerase chain reaction (RT-PCR) on a validated QuantStudio™ 5 Real-Time PCR System (ThermoFisher Scientific, Waltham, MA, USA), with the following primers and probe (Pan-RSV): forward, 5′ GGCAAATATGGAAACATACGTGAA-3′; reverse, 5′-TCTTTTTCTAGGACATTGTAYTGAACAG-3′; probe, 5′-FAM-CTGTGTATGTGGAGCCTTCGTGAAGCT-BHQ-3′ [9]. The lower limit of detection (LOD) was calculated to be 10 genome copies per reaction.

Epidemiological data about the RSV for the area of Larissa were acquired from the National Public Health Organization through the primary health care sentinel surveillance system implemented across Greece. This system collects information from selected physicians (general practitioners, internists and pediatricians) that examine patients in primary health care units, such as health care centers and private medical offices. The selection of sites has been based on a representative sample from all over Greece. Proportional morbidity indices on ILI were obtained for age groups 0–14 and 15+ years of age. Statistical procedures were carried out with the R programing language. To preserve the underlying structure of wastewater data while smoothing out statistical noise, a spline smoothing method was initially applied to RSV and SARS-CoV-2 viral load time series. Next, weekly average concentrations were calculated and analyzed against the proportion of ILI visits. To investigate the possible lagged relationships, initially, Spearman rank correlation coefficients were calculated for lag times of 0, 1 and 2 weeks. Subsequently, a first degree of differencing was applied to both wastewater viral load and proportional notification data to account for autocorrelation. Ljung–Box test and Autocorrelation Function (ACF) plots were used to test for serial correlation. Univariate linear models were run to investigate the association between weekly changes in the viruses’ levels in sewage and respective weekly changes in the frequency of ILI per 1000 visits. For all analyses, a *p*-value of less than 0.05 was considered statistically significant.

## 3. Results

**Figure 1:**
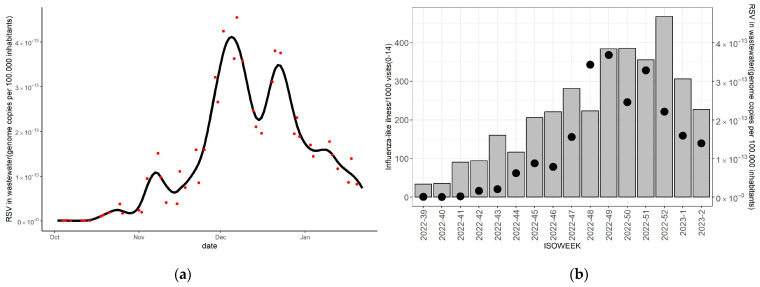
Respiratory syncytial virus wastewater viral load and weekly influenza-like illness (ILI) per 1000 visits in Larissa during the study period. Notes: (**a**) presents the RSV load in wastewater expressed as genome copies per 100,000 inhabitants in the municipality of Larissa (1 October 2022–15 October 2023). Viral load: RNA concentration (Genome copies/L) × Wastewater flow(m^3^/day) × 10^8^/population served by the wastewater treatment plant (WWTP). Red dots represent the viral load corresponding to the date the sample was taken. The Fit curve was generated by spline smoothing. (**b**) presents weekly influenza-like illness (ILI) per 1000 visits from Larissa, in the age group 0–14 years (bars correspond to the left axis). Dots represent the average RSV load for each ISO week and correspond to the right axis.

The first RSV-positive wastewater sample was identified on 18 October 2022 with an RNA concentration of 50 copies/mL. Concentrations gradually increased, peaking on 7 December 2022 (2260 copies/mL), followed by a decline thereafter. Figure 1a presents the actual measurements and the smoothed fit curve of wastewater RSV concentrations during the study period. A similar temporal pattern is observed for the ILI rate per 1000 visits in the age group 0–14 years old, which peaked at week 52 of 2022 (Figure 1b).

The Spearman rank correlation coefficient between weekly RSV viral load and ILI rates in children up to 14 years old was 0.897 (*p* < 0.001). When RSV viral load was lagged by one and two weeks, the correlation coefficients were 0.961 (*p* < 0.001) and 0.924 (*p* < 0.001), respectively. This analysis provides an indication of a strong correlation, especially when wastewater concentrations are lagged by one week. However, caution is suggested since both wastewater RSV and ILI rate data are strongly autocorrelated. To overcome this issue, we explored the relationship between weekly changes (differences) of RSV, SARS-CoV-2 in wastewater samples and proportional notification ILI rate data by applying a first-degree differencing in all time series. For the differenced time series, both the visual evaluation of autocorrelation plots (ACF) and the results of the Ljung–Box test (*p* = 0.838 for RSV viral load, *p* = 0.059 for SARS-CoV-2 viral load, *p* = 0.187 for ILI rate in the 0–14 age group, *p* = 0.771 for ILI rate in the 15+ age group) indicated that the transformed data no longer exhibited autocorrelation.

Next, we ran linear regression models in the differenced time series to depict how weekly changes in wastewater viral load were associated with weekly changes in ILI rates. Table 1 presents the results of the linear regression analysis. Independent variables included RSV and SARS-CoV-2 viral load lagged by 0, 1 and 2 weeks and dependent variables consisted of the weekly changes of ILI rates in age groups 0–14 and 15+ years of age.

For the age group of children younger than 15 years old, the weekly change in RSV viral load at a 1-week lag was strongly associated with the weekly change in ILI rate. No associations were observed when a 0 and 2 lag time was applied and when SARS-CoV-2 viral load was used as a predictor. On the contrary, a marginally statistically significant association was observed between SARS-CoV-2 at a 1-week lag and ILI rate in the 15+ age group.

## 4. Discussion

Our study provided evidence of a significant correlation between the weekly change in RSV viral load in wastewater and the influenza-like illness (ILI) rate per 1000 visits in children younger than 15 years old. In the age group of people older than 14 years old, weekly changes in SARS-CoV-2 viral load were marginally associated with ILI rates. These findings suggest that RSV circulation pattern correlated with ILI rate in young children for whom the ILI rates were higher than adults across the country. Specifically, at the national level, the ILI rate at the peak was approximately 350 cases per 1000 visits for children 0–14 years old compared to 180 cases and 100 cases per 1000 visits for ages between 15 and 64 and older than 64 years old, respectively [10]. On the other hand, a weak association was found between SARS-CoV-2 weekly change pattern and ILI rates among people older than 14 years old, while RSV load was not associated with ILI in this age group. It should be noted that older children and adults with respiratory symptoms are less likely to attend primary health care units compared with younger children. This potential bias could have led to an underestimation of the ILI rates in the 15+ age group but is unlikely to have had a significant impact on the weekly changes since it is considered to be consistent over time. The course of the RSV outbreak in the municipality of Larissa in the autumn and winter of 2022–2023 was captured by wastewater surveillance, indicating that a virus surge started in October 2022 and peaked in December 2022, followed by a gradual decline. In Greece, RSV outbreaks usually occur from December to April, with the highest monthly incidence to occurring in February [11]. However, from the previous year (2021–2022), the epidemic wave started earlier (in September 2021) than in previous years, presumably due to low circulation of RSV during the first year of the COVID-19 pandemic that probably caused an attenuation of the immunity to this pathogen [12]. As the annual seasonal patterns of RSV outbreaks may vary, WBS offers a tool for the timely detection of the RSV season onset and the identification of out-of-season circulation of the virus.

To our knowledge, few studies have examined the potential association between RSV levels in wastewater and epidemiological indicators collected from different surveillance systems. A recent study found that RSV RNA can be detected in settled solids (the matter in wastewater not remaining suspended and precipitates) and reported a strong association between RSV concentrations and positivity rates for RSV at two sentinel laboratories in California, United States [13]. Similarly, in a study conducted in Queensland, Australia, a significant correlation between RSV concentrations and the number of clinical cases was reported [14]. These studies provided evidence about the feasibility and benefits of WBS for RSV monitoring. Our study supports previous findings but also expands the knowledge of the field. Correlation analysis shown similar underlying temporal patterns of RSV and ILI rates in children younger than 15 years old, as shown by the high correlation coefficients. It is interesting that after differencing, a significant association was identified only when RSV viral load was lagged by one week. This finding is important as it indicates that weekly changes in viral load can precede changes in ILI rates in children one week ahead.

Some limitations have to be considered when interpreting the results of the present study. Influenza-like illness can be caused by several pathogens, and thus the investigated association is obviously affected by the circulation of SARS-CoV-2, influenza, rhinoviruses and adenoviruses [15]. Our data indicated that for the particular period and setting, SARS-CoV-2 wastewater levels were a less important determinant of ILI rates in children than RSV and were only associated with ILI in the 15+ age group.

However, the absence of robust evidence on influenza circulation in Larissa is a limitation of the study. On the other hand, national surveillance suggests a high circulation of influenza, especially among young children. Nonetheless, the statistical association between RSV viral load and ILI rate in children was notably strong, suggesting that during RSV outbreaks, WBS can play a key role in the monitoring of the virus’s spread and enhance preparedness, especially in pediatric populations. Another limitation of the present study is that it covers a limited time period.

WBS systems, which are operating in many countries for monitoring SARS-CoV-2, offer a favorable opportunity for incorporating RSV monitoring in sewage from a technical standpoint. This is because there is no requirement for additional wastewater sampling, transportation, storage, or sample pre-treatment. With regard to RSV surveillance, wastewater monitoring can focus on specific time periods when outbreaks are expected or when indications of out-of-season occurrences arise.

Future efforts should also test the validity of systematic monitoring of a panel of respiratory viruses in wastewater to obtain a more comprehensive picture of circulating respiratory pathogens.

## 5. Conclusions

The application of wastewater-based surveillance for RSV can provide useful information on the virus circulation at community level. Our findings suggest that integrating RSV monitoring into current wastewater-based surveillance systems can be highly beneficial.

## Figures and Tables

**Table 1:** Linear regression analysis to determine the association between weekly changes in RSV and SARS-CoV-2 wastewater viral load ^1^ and weekly changes of influenza-like illness (ILI).

	Dependent Variable: Weekly Change of Influenza-like Illness (ILI) Rate per 1000 Visits in Children Younger than 15 Years Old				
Predictors	Lag Time	Std. Beta	95% CI	*p*	R^2^
Weekly change in RSV viral load in sewage (genome copies/100,000 inhabitants)	0 weeks	−0.07	−0.67–0.53	0.799	0.005
	1 week	0.73	0.31–1.14	0.002	0.527
	2 weeks	−0.08	−0.68–0.52	0.774	0.007
Weekly change in SARS-CoV-2 viral load in sewage (genome copies/100,000 inhabitants)	0 weeks	−0.12	−0.72–0.47	0.662	0.015
	1 week	0.28	−0.30–0.85	0.313	0.078
	2 weeks	−0.31	−0.88–0.26	0.258	0.097
	Dependent Variable: Weekly Change of Influenza-like Illness (ILI) Rate per 1000 Visits in Persons Older than 14 Years Old				
Predictors	Lag Time	Std. Beta ^1^	95% CI	*p*	R^2^
Weekly change in RSV viral load in sewage (genome copies/100,000 inhabitants)	0 weeks	−0.17	−0.76–0.42	0.540	0.030
	1 week	0.39	−0.16–0.94	0.149	0.153
	2 weeks	−0.01	−0.61–0.59	0.981	0.000
Weekly change in SARS-CoV-2 viral load in sewage (genome copies/100,000 inhabitants)	0 weeks	−0.35	−0.91–0.21	0.198	0.124
	1 week	0.56	0.06–1.05	0.032	0.308
	2 weeks	−0.10	−0.70–0.49	0.719	0.010

^1^: Viral load: RNA concentration (Genome copies/L) × Wastewater flow(m^3^/day) × 10^8^/population served by the wastewater treatment plant (WWTP) std. Beta: standardized beta coefficient, 95% CI: 95% confidence interval, R^2^: coefficient of determination.

## Data Availability

Data can be provided upon request.
